# Robot-Assisted Complex Anatomical Segmentectomy Using Resection Process Map

**DOI:** 10.1016/j.atssr.2024.06.019

**Published:** 2024-07-04

**Authors:** Shota Nakamura, Megumi Nakao, Shoji Okado, Yuka Kadomatsu, Harushi Ueno, Toyofumi Fengshi Chen-Yoshikawa

**Affiliations:** 1 Department of Thoracic Surgery, Nagoya University Graduate School of Medicine, Nagoya, Japan; 2 Department of Human Health Sciences, Graduate School of Medicine, Kyoto University, Kyoto, Japan

## Abstract

We present a case of robot-assisted complex anatomical segmentectomy utilizing Resection Process Map (RPM) software. RPM enables the confirmation of internal structures obscured by lung parenchyma, thereby reducing the risk of injury or misidentification to essential structures. It facilitates an accurate understanding of anatomy beyond processed vessels, fostering collaboration among the surgical team and informed discussions. Preventing complications stemming from misidentification of bronchovascular structures is vital in complex segmentectomies. Robot-assisted complex segmentectomies can achieve precise anatomical resections through RPM's integration of real-time observations and virtual images. We are committed to further advancing this technology soon.

Pulmonary anatomical segmentectomy has demonstrated noninferior overall survival outcomes over lobectomy for early-stage peripheral lung cancer.^1^ Minimally invasive surgical techniques, such as robot-assisted thoracic surgery (RATS), have gained popularity for performing anatomic segmentectomy.^2^ These methods involve smaller incisions that provide a magnified view of the surgical field. However, the magnified surgical view can occasionally cause misidentification of important structures.^3^^,^^4^ Preoperative computed tomography (CT) is crucial for assessing 3-dimensional (3D) structures due to the considerable variability in vessels and bronchial anatomy.^5^ Current 3D-CT is useful for understanding lung structure, but it cannot consider dynamic changes occurring during intraoperative lung mobilization and ventilation. To address this limitation, Chen-Yoshikawa and associates^6^ developed Resection Process Map (RPM) software, which creates virtual dynamic images based on patient-specific CT scans. Here, we used RPM to assist in a RATS complex anatomical segmentectomy with vascular processing and verification, enabling safe implementation.

### Ethics Statement and Informed Consent

The institutional review board of Nagoya University School of Medicine approved the study protocol (2015-04587082), and the date of approval was March 8, 2016. The patient provided informed consent.

### Concept of the RPM Virtual Image

Previously, we reported the methodology for 3D RPM image generation.^7^ Surface data for each patient’s pulmonary segments, including the targeted resection area, bronchi, pulmonary arteries, and veins, were extracted from Vincent (SYNAPSE VINCENT, Fuji Film Co, Ltd). This surface data was then converted into deformable 3D images using graphic processing unit for each patient. RPM images were developed from contrast-enhanced CT scans and stored in a dedicated computer. The advantage of using RPM is that surgical operations can be conducted while confirming the internal structures. Furthermore, the structures that remain covered by lung parenchyma can be identified in advance, thereby reducing the risk of injury to essential structures.

### Surgical Procedures

The console surgeon controlled the Da Vinci Xi system (Intuitive) from the surgeon console. A single thoracic surgeon performed intraoperative RPM manipulation. A graphical processing unit-equipped computer, connected to all monitors and the surgeon console, enabled the entire operating room staff to share the fused vision of RPM images and real surgical field. The Tile Pro function of the Da Vinci surgical system that displays the actual surgical image at the top and the RPM image at the bottom was used. RPM was visible from the start of the operation until specimen extraction. The RPM operator synchronized the images with the surgeon’s view or as indicated by the console surgeon.

A 66-year-old woman was referred for surgical intervention due to an increased size of multiple ground-glass opacity lesions during follow-up. CT imaging detected a 0.8-cm lesion in the right lung S2, 2.6 cm between the right lung S9 and S10, and a 1.7-cm nodule in the upper lobe of the left lung. The diagnosis was cT1c(3)N0M0 stage IA3. We planned a RATS right S2 and S9 + 10 anatomical segmentectomies. Intraoperatively, the Da Vinci Surgical system’s Tile Pro function was used to display RPM (virtual images) on the bottom of the monitors, enabling vascular processing under RPM guidance (Video 1). The RPM operator ensured the matching of the virtual image on RPM and the actual surgical field in terms of vascular anatomy. We first performed an S2 segmentectomy. RPM simultaneously showed A2b artery removal and underlying bronchus visibility when the surgeon processed this artery, which mirrored the actual surgical view. Indocyanine green was used to delineate the segment’s boundary, thereby accomplishing the S2 segmentectomy. Subsequently, we performed S9 + S10 segmentectomy. The RPM operator ensured that the RPM image stayed synchronized with the actual view, as the surgeon dissected the inferior pulmonary artery, to confirm that the artery was indeed A9 + 10. The underlying bronchus in the RPM image was revealed on the RPM operator’s removal of the processed A9 + 10 on the virtual image. The surgeon followed RPM’s synchronized guidance to identify B9 + 10. RPM simultaneously removed B9 + 10, revealing the course of the adjacent vein, when the surgeon processed the bronchus. The surgeon identified and managed the veins to preserve V6 and V7 + V8 and process V9 + V10, as further confirmed by RPM and the actual view. Using indocyanine green delineation, we accomplished the anatomically complex segmentectomy using RPM. The patient was discharged without any adverse events on postoperative day 6 and was able to receive pulmonary resection for the contralateral nodule after 1.5 months.

## Comment

Herein, we present a case of RATS complex anatomical segmentectomies using RPM. Precise comprehension of vascular structures, particularly veins, is of paramount importance pertaining to complex segmentectomy. Errors in identifying and managing these veins may cause unintended larger pulmonary resections or even lobectomy. RPM use in complex segmentectomies enables the surgeons to accurately determine anatomy beyond processed vessels. It helps collaborate among the surgical team by enabling real-time observations to be compared with virtual images, resulting in informed discussions and consensus on accurate vascular processing. Current commercial navigation systems cannot synchronize the removal of processed vessels in virtual images with the detailed visualization of structures beyond them, as achieved with RPM. The effectiveness of RPM has introduced a new safety and efficacy level in contemporary surgical practice.

However, the navigation systems present several challenges, as highlighted in this report. Currently, RPM-based surgical navigation requires manual synchronization by thoracic surgeons, who have a comprehensive understanding of anatomical knowledge and surgical experience. The RPM operator aligns the virtual image on the RPM with the actual surgical field, focusing on pulmonary vessels and the degree of lobar separation. The underlying bronchus in the RPM image becomes visible when the RPM operator removes the processed vessel from the virtual image. The surgeon uses this synchronized RPM guidance to identify and dissect the bronchus or other pulmonary vessels. We are in the process of developing a system for automatic synchronization of the RPM with the actual surgical image, aiming to implement it in the near future.

In conclusion, preventing complications stemming from the misidentification of vascular structures is crucial in complex segmentectomies. A thorough understanding of anatomy is essential for success. RATS segmentectomies can achieve precise anatomical resections through the integration of real-time observations and RPM’s virtual images, which are collaboratively used by the entire surgical team. We are committed to further advancing this technology soon.
